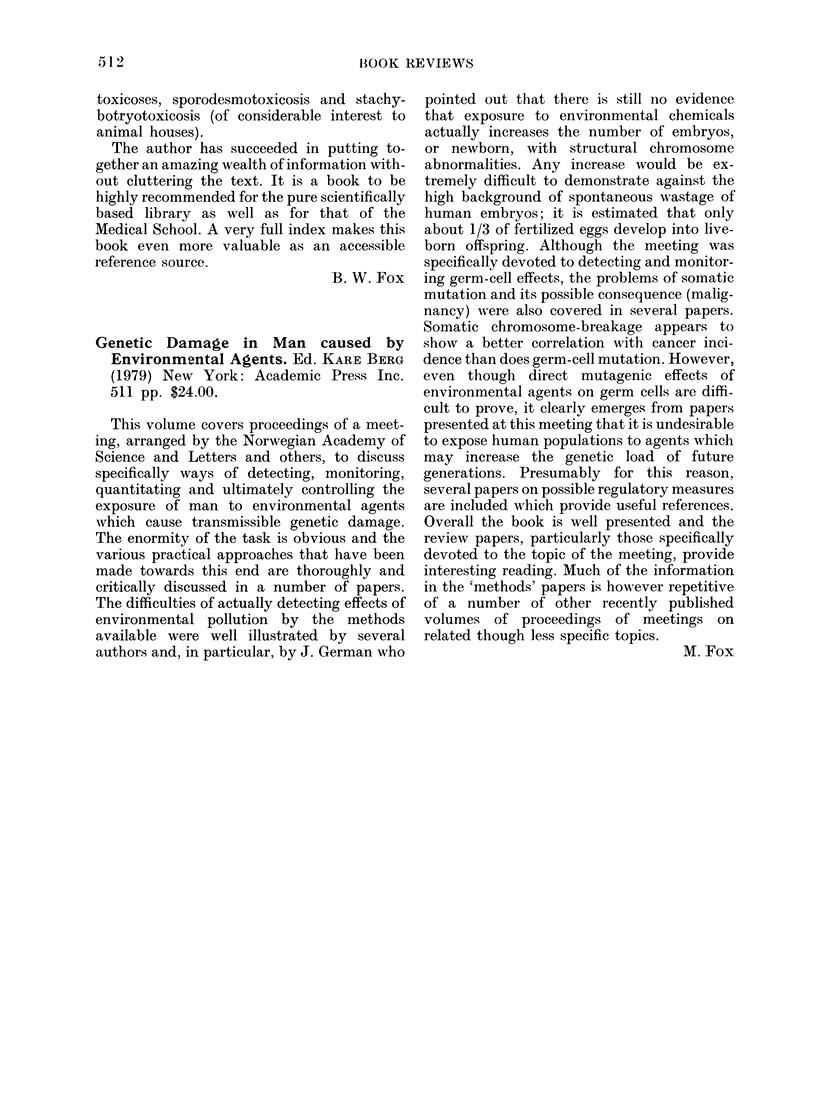# Genetic Damage in Man caused by Environmental Agents

**Published:** 1980-03

**Authors:** M. Fox


**Genetic Damage in Man caused by Environmental Agents.** Ed. Kare Berg (1979) New York: Academic Press Inc. 511 pp. $24.00.

This volume covers proceedings of a meeting, arranged by the Norwegian Academy of Science and Letters and others, to discuss specifically ways of detecting, monitoring, quantitating and ultimately controlling the exposure of man to environmental agents which cause transmissible genetic damage. The enormity of the task is obvious and the various practical approaches that have been made towards this end are thoroughly and critically discussed in a number of papers. The difficulties of actually detecting effects of environmental pollution by the methods available were well illustrated by several authors and, in particular, by J. German who pointed out that there is still no evidence that exposure to environmental chemicals actually increases the number of embryos, or newborn, with structural chromosome abnormalities. Any increase would be extremely difficult to demonstrate against the high background of spontaneous wastage of human embryos; it is estimated that only about 1/3 of fertilized eggs develop into liveborn offspring. Although the meeting was specifically devoted to detecting and monitoring germ-cell effects, the problems of somatic mutation and its possible consequence (malignancy) were also covered in several papers. Somatic chromosome-breakage appears to show a better correlation with cancer incidence than does germ-cell mutation. However, even though direct mutagenic effects of environmental agents on germ cells are difficult to prove, it clearly emerges from papers presented at this meeting that it is undesirable to expose human populations to agents which may increase the genetic load of future generations. Presumably for this reason, several papers on possible regulatory measures are included which provide useful references. Overall the book is well presented and the review papers, particularly those specifically devoted to the topic of the meeting, provide interesting reading. Much of the information in the 'methods' papers is however repetitive of a number of other recently published volumes of proceedings of meetings on related though less specific topics.

M. Fox